# Effect of a Canine-Assisted Read Aloud Intervention on Reading Ability and Physiological Response: A Pilot Study

**DOI:** 10.3390/ani9080474

**Published:** 2019-07-24

**Authors:** Suk-Chun Fung

**Affiliations:** Department of Special Education and Counselling, Education University of Hong Kong, 10 Lo Ping Road, Tai Po, Hong Kong; fungsc@eduhk.hk; Tel.: +852-2948-7789

**Keywords:** animal-assisted education, canine-assisted read-aloud, heart rate variability, reading accuracy, reading fluency

## Abstract

**Simple Summary:**

Canine-assisted reading aloud programs have been attracting growing interest because reading to a dog may contribute to the reading performance of children. The positive effect of a canine-assisted reading aloud program, however, received limited empirical support. Specifically, most of the current papers were papers of expert opinions. The current pilot study aimed at providing preliminary evidence for the effects of reading to dogs on three lower-performing third-graders’ reading abilities and understanding their physiological stress responses. The findings of the study implied that canine-assisted reading aloud programs had potential to increase the reading fluency and relaxation level of children with lower performance when reading to a dog.

**Abstract:**

The aim of this study was to determine whether there is an increase in the reading fluency and accuracy of three lower performing third-graders after participating in a canine-assisted read-aloud program, as well as an increase in the relaxation level during and after the program. This study employed a pre-test-post-test design to test the hypotheses that gains would be made in both reading fluency and reading accuracy upon completion of the program. The three grade 3 students were assessed by the Chinese Character Reading Test and the Reading Fluency Test. During the intervention, they read to a trained canine in the presence of a handler. Three days after the completion of the seven 20-min interventions, the participants were assessed by the two standardized reading tests a second time. Heart rate variability (HRV) responses to the pre-test, the intervention and the post-test were recorded. The three grade 3 students attained a higher level of relaxation while reading to the dog and increased their reading fluency after the reading sessions. These results provided preliminary evidence that the canine-assisted read-aloud program can increase the reading performance of children with lower performance. Implications for future research and reading programs will be discussed.

## 1. Introduction

Companion animals could be beneficial to school children in many ways. In recent decades, a growing body of evidence has shown the positive effects of animal-assisted education (AAE), which is a form of animal-assisted intervention (AAI), in enhancing humane values [1,2] and reading skills and attitudes [3,4,5]. Amongst the AAE programs, canine-assisted read-aloud programs seem to have become the most prevalent ones in schools in the past two decades [6,7]. A canine-assisted read-aloud program is actually a visitation program in which a handler brings a trained therapy dog to school to work with students for reading aloud. Reading education assistance dogs (READ), which is one of the first canine-assisted reading programs, was first launched in Salt Lake City in 1999. Currently, READ is being practiced in many states of the US and has new chapters in many countries, such as Germany, Italy, Netherlands, Norway and Sweden. READ has also been replicated in many English-speaking countries, for example, the Bark and Read Foundation in UK, Caring Canines in US, Dogs Helping Kids in UK, Read2Dogs in UK, SitStayRead in US, Reading with Rover in US, All Ears Reading in US, and Classroom Canines in Australia.

There are four key rationales for the prevalence of canine-assisted read-aloud programs. First, school reading programs are usually strongly supported by the government because of the high economic and social costs of illiteracy. According to the findings of the final report of the World Literacy Foundation [8], the cost of illiteracy to the global economy is estimated at USD $1.2 trillion. The social cost of illiteracy is huge. Illiteracy has a clear link with poor health, crime, and being welfare dependent [8]. Literacy does have an important role to access to employment [9,10] and break inter-generational cycles of under-achievement [11]. Literate people can also acquire better nutritional knowledge and preventive health measures, as well as demonstrate better hygiene practices [8]. Those people who have strong literacy skills are valuable human capital to their nation. It is therefore easy to understand why it is largely uncontroversial for governments to support and fund school reading programs.

Second, reading programs, particularly innovative ones, are very likely welcome by parents and schools. Good reading skills can not only enable language and mathematics acquisition but also enable educational attainment [7,11,12,13]. Improving reading skills is a key first step in attaining a higher level of literacy, encouraging the completion of a higher education degree and ensuring the next generation is not trapped in a cycle of poverty and disadvantage [8]. While the importance of enhanced reading skills is known, it is not an easy task to enhance reading motivation and enjoyment. It was found that reading enjoyment is more important for children’s educational success than their family’s socio-economic status [14]. Both parents and teachers are eager to find reading programs that can engage children in reading and thus enhance children’s reading abilities. To date, interest in reading aloud to dogs programs has attracted numerous media coverage. The pictures of children who are reading story books to dogs are usually impressive and convincing. Reading to a dog was also documented to be effective intervention to increase reading motivation and confidence as well as reduce reading anxiety of the children [15,16,17]. Though there must be some parents and school administrators still have great hesitations to implement any learning activities with dogs, canine-assisted reading programs, such as READ and SitStayRead, have become more recognized by parents and teachers.

Third, canine-assisted read-aloud programs are manageable by community reading teams as volunteer handlers with little training are eligible to participate in the program. For example, in the READ [18] Team Training Manual, it is mentioned that the READ teams are all volunteers and therefore the running cost of the program is minimum. Given that the program requires limited professional human resources, it may represent a unique, cost effective intervention that could be implemented into a wide scope.

Finally, canine-assisted read-aloud programs are usually welcome by school children. It is an innovative and funny experience to read aloud to a well-trained dog in school. During the reading process, the trained canine is a non-judgmental audience who does not comment on or interfere with children’s reading performance. It is a comforting companion who can help build a safe and warm environment for children to read aloud [6]. To children, reading to a calm, non-judgmental and well-trained canine companion might make the reading process and practice less stressful [15,16,17] and even more enjoyable [19].

The support of all stakeholders, including governments, schools, parents, reading teams and students provide sufficient conditions for the prevalence of the canine-assisted read-aloud program in schools in many countries. Currently, empirical evidence is showing the effectiveness of canine-assisted read-aloud intervention. In a recent systematic review, 48 studies of canine-assisted reading programs demonstrated positive effects of the children reading to dogs [7]. The effects of reading aloud to dogs have been evaluated in two dimensions: The improvements in actual reading abilities and the changes to behavioral processes. According to the review paper [7], only around one-third of the reviewed studies examined the effects of canine-assisted read aloud programs on reading abilities. These studies are mostly papers of experts’ opinions [15,16]. There are a few studies using standardized tests to measure reading accuracy, fluency and comprehension, such as the studies of Fisher and Cozen [20] and Le Roux, Swartz and Swart [4]. Fisher and Cozen [20] used the Neale Analysis of Reading Ability (NARA) to assess an eight-week reading to a dog program and reported a disengaged reader improved in reading accuracy at the completion of the program [20]. Le Roux, Swartz and Swart [4] also used NARA to evaluate a 10-week canine-assisted reading program for poor third grade readers. As a result, both reading accuracy and reading comprehension were better in the dog group compared to the adult group, teddy bear group and control group. It was also found that reading rate was significantly better in the dog group compared to the teddy bear group [4]. While the two studies clearly tell brief canine-assisted read aloud programs could have positive effects on reading abilities, they are the minorities in the field. More research in this direction is needed.

Currently, the majority of evaluation studies of canine-assisted read aloud interventions were set out to investigate the impact of reading to a dog on the behavioral and emotional processes. Specifically, around two-third of the reviewed papers investigated the effects on reading motivation, reading confidence, reading enjoyment, reading engagement, feelings of support, self-esteem, anxiety and stress [7]. Again, most of these studies are opinion papers of classroom teachers and dog handlers e.g., [15,17,21]. Behavioral measures have been utilized in a few studies of this line, e.g., the elementary reading attitudes scale (ERAS) in Konarski’s study [22] and the behavioral observation of students in schools (BOSS) in Bassette’s study [23]. There is one study using a physiological measure to assess the effects of reading on the anxiety levels of 38 children when a dog was present. It was found that the presence of the dog resulted in lower blood pressures both while the children were resting and while they were reading. However, the study was actually experimental in nature. The children did not touch or talk to the dog during the one-session experiment [24].

In short, the effectiveness of canine-assisted read-aloud programs has yet to be verified in full because of the poor quality of the evidence. In view of the current status, there is a clear need to establish more evidence on the effects of reading to dogs by using standardized tests to directly measure the actual reading abilities. Evidence from physiological indicators of behavioral and emotional processes would be most desirable. Furthermore, it is also necessary to continue research in different cultures [4]. Cultural differences in children behavior and perceptions with animals may be an important issue that warrants careful consideration in both practice and experiential design. For example, putting oneself in the limelight for individual praise is not considered appropriate in some cultures. As such, children may exhibit discomfort or embarrassment at being singled out for special attention [18]. In addition, children in some cultures may associate reading aloud with memorization while others may see reading as an essential oral activity and will read aloud spontaneously [18]. At the same time, children’s perceptions with animals in different cultures may affect children’s motivation to interact with animals. Those children with less positive perceptions with animals are less likely to show improvements through animal interactions [25].

The present study is the first evaluative study of a canine-assisted read-aloud program conducted for Hong Kong Chinese students. In Hong Kong, children are taught Chinese as their first language and English as their second language in formal education. School curriculum is packed with numerous subjects and intensive homework as well as assessment. School learning in Hong Kong can be best described as busy and rushed. In terms of language learning, Hong Kong students learn to read Chinese characters using the look-and-say method without a system of phonetic symbols to label their pronunciations [26]. However, reading aloud practice has never been a priority in language classes, particularly in Chinese language classes. The language learning context in Hong Kong seems particularly difficult for students who are low performing in reading. As stated by Ecklund and Lamon [12], reading motivation may be especially important for these students as poor reading abilities are associated with low reading motivation. Reading anxiety is also commonly seen in below-average readers [27]. Reading to dogs seems to be particularly beneficial to poorly performing readers in terms of an increase in reading motivation and a decrease in reading anxiety. This pilot study set out to investigate if reading to a dog could enhance the reading abilities of three Hong Kong Chinese students who were low performing in reading. Standardized tests were used to measure reading fluency and accuracy before and after the canine-assisted reading aloud program.

The present study is also the first evaluative study of a canine-assisted read-aloud program measuring physiological responses of children. Specifically, heart rate variability (HRV) responses to pre-test, to the program and to post-test were recorded to measure the real time physiological stress responses of the children to reading. While a physiological measure of anxiety would have the advantage of being free from experimenter bias, the comfortable level of the participants being measured was also an important consideration in this pilot study. Considering that most children are learning to read up until the end of third grade [28] and they could independently attend individual intervention sessions, this pilot study finally selected third graders as participants, just like most of the current reading to dog programs did e.g., [4,23,29,30,31].

In short, the current pilot study set out to evaluate a brief reading to dog program for three Hong Kong Chinese third-graders with low performing in reading. It aimed to determine: (1) Whether there is an increase in the reading fluency of the poorly performing third-graders after participating in the program; (2) whether there is an increase in the reading accuracy of poorly performing third-graders after participating in the program and (3) whether there is an increase in the relaxation level of the poorly performing third-graders during and after participating the program.

## 2. Materials and Methods

### 2.1. Participants

The present study was conducted in a mainstream primary school in Hong Kong. As the study used a single-subject experimental design, the author asked the teachers to nominate a small number of grade three students to participate in a screening reading test. A group of fifteen grade three Chinese-speaking students, two girls and 13 boys, were selected by the school teachers as the most in need of additional assistance in improving their reading performance. Teachers’ nominations were based on their experience and acquaintance with these fifteen students and their performance in school. Parental consent was obtained before the start of the screening test. The fifteen grade three students were then assessed by a test that comprised of a 2-min Chinese Character Reading Test and a 1-min Reading Fluency Test (see Section 2.7. Assessments).

**Figure 1:**
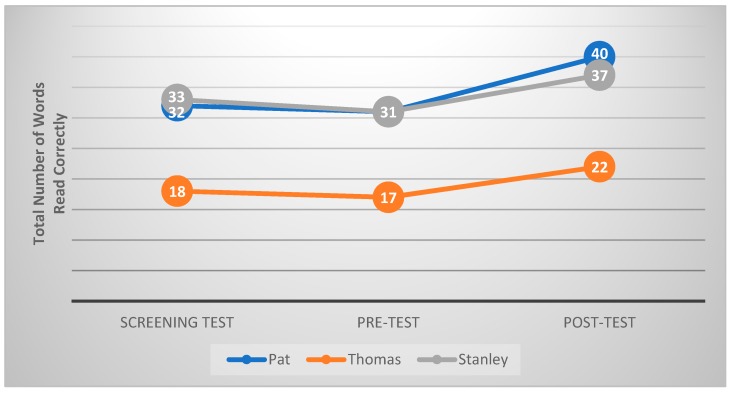
The reading speed by each child within 45 s across all time points.

**Figure 2:**
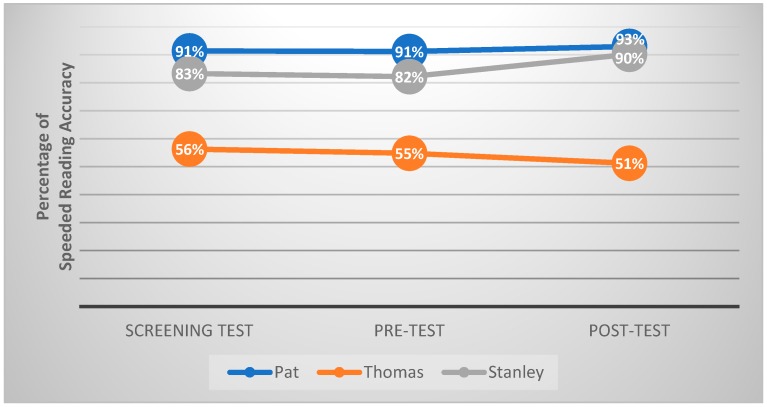
The speeded reading accuracy by each child within 45 s across all time points.

**Figure 3:**
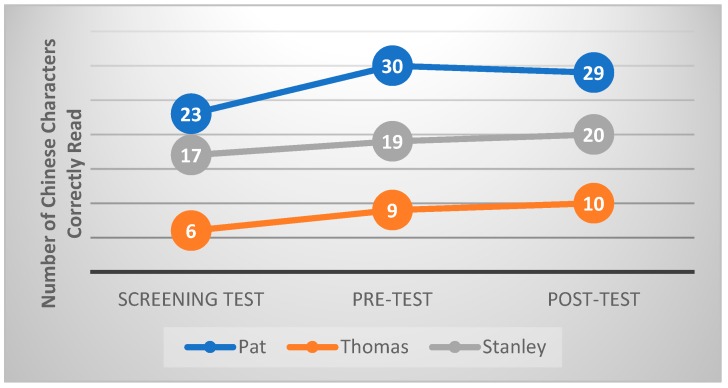
The characters read correctly by each child across all time points.

Three students with the lowest performance in the screening test were invited to participate in the present study. Pat was an eight-year-old girl and Thomas and Stanley were eight-year-old boys. Figure 1, Figure 2 and Figure 3 show the results of the screening test for Pat, Thomas and Stanley. Prior to the start of the study, their parents had given written informed consent to the terms of the study and confirmed that they had no pet at home. The parents also completed the client screening form for animal assisted therapy to affirm their children’s suitability for using a trained canine in the reading sessions. The client screening form was developed by Chandler [32] and was translated by the author into Chinese and utilized in a previous study of animal-assisted play therapy [33]. Examples of the 13 screening items included ‘‘Does the client have animal allergies? Which animals?’’, “Does the client have animal fears or phobias? Which animals?” and ‘‘Does the client have a history of aggression or abuse toward animals?’’. All participants were considered to be appropriate for the intervention. No concerns were raised for any participants.

### 2.2. Canine Team

A certified therapy dog, Pearl Pearl, and her handler from the Hong Kong Animal-assisted Therapy Association (HKAATA), a non-profit organization organizing animal-assisted therapy (AAT) and animal-assisted education (AAE), were recruited to the reading program. The dog and the handler were assessed and certified as a Certified Therapeutic Canine Team by the HKAATA. Pearl Pearl was a five-year-old female toy poodle. The handler, who was a registered social worker and the founder of the HKAATA, was a certified AAT therapist of the Professional Animal-Assisted Therapy Association of Taiwan.

### 2.3. The Intervention: Canine-Assisted Reading Aloud Program

The current canine-assisted reading aloud program, which was a form of AAE, was a reading program that incorporated a trained, calm canine into planned reading aloud sessions. Similar to other reading to dog studies e.g., [4,20], this study employed a short and intensive approach. The twice a week, eight-session program focused on creating a context of acceptance in which children with poor reading performance could practice their reading skills without fear of making mistakes. In the program, the child was a storyteller who read aloud stories to the canine. The canine was a non-judgmental audience and a comforting companion for the child. The dog handler acted as an active listener [6]. In each 20-min session, the first five minutes were scheduled as free time for interactions between the child, the dog and the handler. The handler began the 15-min reading time with these instructions: “Today, Pearl Pearl, you and I have a special reading time. It is time for you to read stories to Pearl Pearl. If you don’t know how to read a word, you could ask me how to read, or try to read it, or just skip the word. Remember! It is no problem to read it incorrectly. It is fine. Do you have any questions? (a pause) We have 15 min for the reading time. Let’s start now!” During the reading sessions, the handler only provided feedback or reading assistance if specifically asked by the child.

### 2.4. Reading Materials

The three participants were allowed to bring their favorite Chinese storybooks to the reading sessions in order to enhance their comfort level. Additionally, they could choose any Chinese storybooks from the school library prior to each session.

### 2.5. Setting

All reading sessions were conducted in a private corner of approximately 5.6 m^2^ in the school library. In each session, one child sat next to Pearl Pearl. The handler also sat near Pearl Pearl to help ensure Pearl Pearl sat or laid quietly while the child read. The screening test, pre-test and post-test for each individual child were conducted in a classroom of the school.

### 2.6. Study Design

A pre-test–post-test design was used to evaluate the effects of the reading aloud to dog intervention program on three Chinese-language grade three students who were lower performers in reading. After a screening test for 15 third-graders, the three lowest performers were invited to participate in the current study. The assessment consisted of two dimensions of measures. The first dimension was the measure of reading abilities. Two standardized reading tests, (1) the Chinese Character Reading Test and (2) the Reading Fluency Test, were conducted in the pre-test. During interventions, each participant attended eight reading to dog sessions, 20 min each, twice a week, which were conducted in the school library during their extra-curricular activity time period. Three days after the completion of the four-week intervention, each participant was re-assessed with the same pre-test measures described above. The second dimension was a real time measure of physiological stress responses to reading. HRV responses were recorded. HRV was measured with a pulse signal obtained from a photoplethysmography sensor attached to the earlobe and monitored in the pre-test, each 20-min reading session and post-test for each participant.

### 2.7. Assessments

#### 2.7.1. Reading Fluency 

The Reading Fluency Measure is a timed reading task to measure reading fluency. In this measure, there are two four-character words, five three-character words, 76 two-character words and 21 single-character words for a total of 104 Chinese words. Prior to the testing, the subject was given eight practice words. In this task, the subject was required to read as many Chinese words as possible within 45 s to obtain a score for reading speed (the total number of correctly read words). This score was used to represent the level of reading fluency. The measure has been used in the study of Pasquarella et al. [34]. The internal consistency reliability (Cronbach’s Alpha) for this task was .90.

#### 2.7.2. Reading Accuracy

The Reading Fluency Measure was also used to measure reading accuracy of the children. In the task, the children were required to read as many Chinese words as possible within 45 s to obtain a score for speeded reading accuracy (divide the number of words read correctly by the total number of words read).

The Chinese Character Reading Test was another tool to measure reading accuracy in this pilot study. In this test, individual subjects must read aloud 100 single Chinese characters. These characters were chosen from various primary school Chinese textbooks. The items were arranged in ascending order of difficulty. One point was given when the subject pronounced the character correctly, while no point was given when the subject gave an incorrect pronunciation. Once the subject failed to recognize 10 characters consecutively, the testing would stop. The measure has been used in other studies [35]. The internal consistency reliability (Cronbach’s Alpha) for this task was .96.

#### 2.7.3. Heart Rate Variability (HRV)

HRV is the fluctuation in the time intervals between adjacent heartbeats and is used in psychophysiology as a stress indicator [36,37]. Higher values of HRV indicate a greater level of relaxation and are also associated with higher prefrontal cortex activity and cognitive demand [38]. RMSSD, which is a standard time dependent measure of HRV, was employed for analysis in the current study. RMSSD refers to the root-mean square of successive differences between normal heartbeats. It is obtained by first calculating each successive time difference between heartbeats in mini seconds. Each of the values is then squared and the result is averaged before the square root of the total is obtained [36]. In the current study, it was measured with a pulse signal obtained using emWave Pro Plus, a publicly available biofeedback device, from a photoplethysmography (PPG) sensor attached to the earlobe of the participants. Via a USB device, the earlobe was connected with a wire to a computer where data collection was monitored real-time by a research assistant.

### 2.8. Procedures

Permission to conduct the study was obtained from the Education University of Hong Kong’s Human Research Ethics Committee (Ref. No. 2017-2018-0339). The parents of all the 15 third graders gave written informed consent for their children’s participation in a screening reading test, which was scheduled in November of 2017. The author then identified the three lowest performers in the screening reading test and invited them to participate in the study. Prior to the intervention, parental consent for participation in the study and video-taping was obtained. In addition, the three third graders passed the client screening assessment. The pre-test was conducted in April of 2018, four days before the implementation of the reading sessions. One of the sessions was cancelled because two participants were unavailable. Finally, the post-test was conducted three days after the completion of the program.

## 3. Results

### 3.1. Reading Fluency

Figure 1 shows the reading speed, which is the total number of words read correctly within 45 s in the Reading Fluency Measure by each participating child in the screening test, pre-test and post-test. The results showed that the canine-assisted read-aloud program produced an increase in reading speed for Pat, Thomas and Stanley of nine words (29%), five words (29%) and six words (19%) respectively, when compared with their pre-test results.

### 3.2. Reading Accuracy

Figure 2 shows the percentage of speeded reading accuracy, which was calculated by dividing the number of words read correctly by the total number of words read within 45 s in the Reading Fluency Measure by each participating child in the screening test, pre-test and post-test. The results showed that the canine-assisted read-aloud program produced an increase in speeded reading accuracy for Pat and Stanley of 1.8% and 8% respectively, when compared with pre-test results. In Thomas’s case, the speeded reading accuracy in the post-test decreased 3.6%, when compared with the pre-test result.

Figure 3 shows the number of Chinese characters correctly read in the Chinese Character Reading Test by each participating child in the screening test, pre-test and post-test. The results showed that the pre-post difference in the number of correctly read characters is one in each of the three children. This result suggests that the intervention did not have any effect on reading accuracy. At the same time, it was found that the number of Chinese characters correctly read in the pre-test increased by 7, 3 and 2 for Pat, Thomas and Stanley respectively, when compared with the screening test results.

### 3.3. HRV

**Figure 4:**
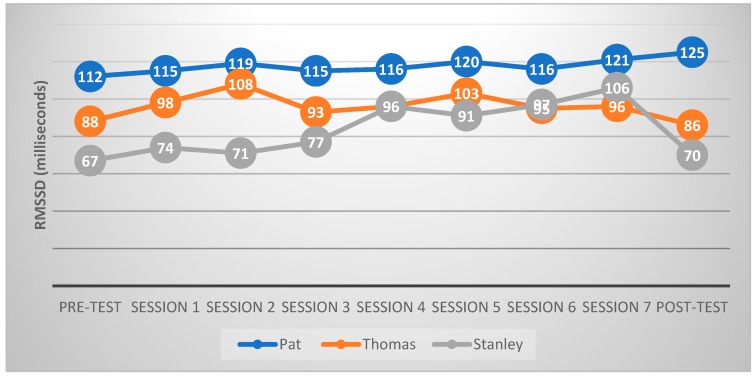
The RMSSD, heart rate variability (HRV) measures, of the three third-graders in the pre-test, during the intervention and in the post-test.

HRV data were analyzed using Kubios HRV Standard software. Data from 2 min to 5 min of the pre-test and post-test period, as well as from 7 min to 10 min of each of the intervention sessions were used in analyses. A medium correction threshold was chosen for artifact correction. Figure 4 shows the HRV measures of each child in the pre-test, during the intervention and in the post-test. Two observations were made. First, the RMSSD increased from 2.7% to 8.0% for Pat, from 5.7% to 22.7% for Thomas, and from 6.0% to 58.2% for Stanley during the seven reading sessions, when compared with the pre-test results. The increased RMSSD in all the three third-graders suggested that their relaxation levels when reading aloud to a dog were higher than those when reading in the pre-test. Second, the RMSSD remained unchanged in the post-test, when compared to the pre-test results for the two boys. This observation suggests that the program did not have any effect on stress reduction during the post-test.

## 4. Discussion

### 4.1. Reading Ability

The present pilot study aimed at evaluating the effectiveness of a canine-assisted read-aloud program on the reading abilities and physiological responses in three third-graders with poor reading performance. With regard to reading abilities, positive findings of the canine-assisted read-aloud program were found, but only in reading fluency. The gains in reading speed shown in this study, which were consistent with the result of the previous studies [4,31], were unlikely to be due to maturation. In this study, the reading speed of the children in the pre-test was nearly the same as that in the screening test, which was conducted five months before the pre-test. It is encouraging that even with a relatively brief intervention (seven 20-min sessions in a month), 19% to 29% gains were found in the reading speed of the three children with poor reading performance. This positive finding is particularly encouraging as oral reading fluency, as concluded in the study of Fuchs et al. [39], can serve as a strong indicator of overall reading competence. Fuchs et al. [39] explained that reading fluency captures individual differences in a number of reading subcomponents at lower and higher levels of processing.

An increase in reading accuracy, however, was not found in the three low-performing third-graders after the program. This result could be explained by the child-centered nature of this program. In the present study, the participating children were given choices. They could choose their favorite story books. They could also choose to skip difficult words, read the words although they were unsure about the correct pronunciation or ask the handler for the correct pronunciations. In this reading program, the handler’s role was not that of a teacher, but of a caregiver of Pearl Pearl. The rationale behind the choices given was to cultivate an accepting reading environment in which the children experienced a higher level of autonomy. In the reading sessions, Pearl Pearl played as a non-judgmental audience. As Fung [6] stated, the trained dog could provide the children with a sense of greater capability and accomplishment. While the child-centered approach further helps to cultivate a relaxing reading aloud environment, it may not be effective to improve reading accuracy in a brief intervention. Specifically, the children were allowed to read story books even if the books were not level appropriate. Without a process of exposure to difficult words and getting feedback about their reading errors, the current program was not able to increase the reading accuracy of the participating children. In contrast, it was found that the reading accuracy increased from the screening test to the pre-test. Maturation appears to be the reason for the increase of reading accuracy during this five-month classroom learning. This result suggests that there is a need for a more intensive intervention that includes intentional instruction in word recognition as well as engaged reading practice with adults’ corrections of reading errors to increase reading accuracy. To gain greater support from the government, schools and parents, the development of a graded canine-assisted read-aloud curriculum that has specific and measurable learning outcomes seems important.

### 4.2. Physiological Response

In this study, HRV analysis was performed to obtain a real-time record of the beat-to-beat changes in the three third-graders’ heart rates as they occurred in response to reading tests and reading aloud to dog interventions. Such investigation will help us to understand the effectiveness of the intervention in enhancing the relaxation level of the children to reading test. In addition, it would help us understand their relaxation level in a reading environment in which a trained dog was a non-judgmental companion. The results of the current study suggested that all the three third-graders were more relaxed throughout the reading aloud to dog sessions than during the pre-test and post-test situations. It is possible that repeatedly practicing reading story books in a relaxing environment with the companion of a trained dog could improve the children’s reading fluency. The relaxation level of both Thomas and Stanley in the post-test, however, returned to the level measured in the pre-test. Only in Pat’s case was the relaxation level observed to have an increasing trend from the pre-test, to the intervention and finally the post-test. In this pilot study, the effect of the reading intervention on the change of physiological responses to test scenarios could not be concluded.

Nevertheless, HRV is considered a measure of neurocardiac function that reflects the complex interactions of the heart with multiple body systems [40]. In addition, HRV monitors autonomic nervous system activity and assesses its reactivity under different conditions [41]. The pulse data were easily collected using emWave Pro Plus with its ear clip. In the current study, all the three children were comfortable with this arrangement. As a physiological measure, HRV is different from blood pressure measurements as it is a real-time measure of a period of time. Analyses can also be easily performed for a selected number of time periods. It is different from ECG devices as PPG sensors and devices have no electrical interaction with the human body. The HRV measure is non-invasive, cheaper and requires less maintenance than the ECG device [42]. The pilot study suggests that the HRV is a suitable choice for collecting biofeedback data in canine-assisted read-aloud programs.

### 4.3. Limitations and Future Studies

In this pilot study, the investigation of both the reading abilities and physiological responses of the participating children provided preliminary evidence regarding the pedagogic value of involving a trained companion dog in a reading aloud program. Future canine-aloud studies should continue to employ standardized tests to demonstrate the effectiveness of the intervention on reading performance, as well as taking physiological measurements, such as HRV, to obtain the real-time relaxation level in the reading to dog sessions. A key limitation deserves our attention, however: The current small sample size did not allow for conducting either statistical tests or randomized controlled trials (RCT). In this pilot study, only three third-graders were included. Though there was a trend of increase in reading fluency measure scores, the numerical increase between the data points could not enable confidence in the detection of meaningful effects. Though actual reading abilities and reading relaxation level were both measured in this pilot study, the small sample size did not allow for running statistical tests to test the inference: “reading to a dog may have a beneficial effect on a number of behavioural processes which contribute to a positive effect on the environment in which reading is practiced, leading to improved reading performance” [7] (p. 1). In addition, although it was found that the relaxation levels of the three children were higher than those of the pre-test and post-test, there was no evidence to illustrate the unique contribution of the trained dog to the cultivation of the relaxing environment. Future studies should increase the sample size to perform statistical analysis of the measures and use of RCT to establish evidence about what effects reading to dogs may have on children’s reading practices. To identify the role of the trained dog, a comparison between a group with a trained dog and a group without a trained dog is necessary. Future studies are also suggested to identify the role of the handler by comparing groups in which the participation level of the handler is different. It is believed that such comparison evaluation would help us identify the approach(es), which is(are) most likely to benefit school children in these programs.

The measures of this study were also limited. Future studies should increase the number of standardized measures. In regard to reading measures, the participants were required to read words in isolation in both the Chinese Character Reading Test and Reading Fluency Measure in the current study. It is suggested that some standardized reading measures can be added to provide more data on reading abilities. As concluded by Jenkins et al. [43], text fluency seems to have more in common with reading comprehension than with reading word lists fluently. As a further index of participants’ reading abilities, standardized reading measures that require reading words in context could be added. In addition, measures of reading motivation, such as the Motivation for Reading Questionnaire (MRQ) and Reading Activity Inventory (RAI), are also suggested for inclusion in future studies.

## 5. Conclusions

The present study adds to the growing body of research on AAE and makes a unique contribution by evaluating canine-assisted read-aloud programs for Chinese-speaking school children in particular. A significant strength of the present pilot study relative to earlier studies was the use of two standardized reading measures with high internal consistency and reliability and HRV, a real-time stress indicator in psychophysiology. The results of the pilot study implied the canine-assisted read-aloud program is a feasible reading practice and has good potential to develop as a clinically and economically effective reading intervention. The next step of the development is to ensure enough of a sample size to conduct statistical analysis of the reading abilities and physiological measures.
